# Editorial: Management of testicular microlithiasis in children

**DOI:** 10.3389/fped.2026.1894440

**Published:** 2026-07-17

**Authors:** Nahla Kechiche, Sonia Pérez-Bertólez

**Affiliations:** 1 Department of Pediatric Surgery, Fattouma Bourguiba Hospital, University of Monastir, Monastir, Tunisia; 2 Pediatric Urology Unit, Pediatric Surgery Department, Hospital Sant Joan de Déu, Universitat de Barcelona, Barcelona, Spain

**Keywords:** cancer, management, microlithiasis, pediatric, testicular

Pediatric testicular microlithiasis (TM) is an uncommon, often incidental ultrasound finding in boys, whose clinical relevance, cancer risk, and optimal follow-up remain intensely debated. The two contributions in this Research Topic provide complementary perspectives: one focused on clinical and imaging diagnosis, the other on epidemiology, risk associations, and management. Together, they move the field toward more standardized, risk-adapted care while underscoring major evidence gaps.

## 1 Definitions, pathology, and imaging

The mini-review by Zitouni et al. refines histological and radiological definitions of TM, emphasizing that microliths are laminated calcifications within seminiferous tubules and that ≥5 hyperechogenic foci <3 mm on ultrasound define classic TM. Goede's classification into classic vs. limited TM and diffuse vs. segmental patterns is highlighted as clinically useful.

Habachi et al. focus on the diagnostic dilemma, proposing an imaging-based algorithm and stressing the central role of high-frequency ultrasound and careful physical examination. High-resolution ultrasound serves as the gold standard for evaluation, offering a radiation-free, cost-effective tool with good inter-observer consistency.

## 2 Epidemiology, associations, and cancer risk

Zitouni et al. synthesize prevalence data, suggesting that TM in children is more frequent than once believed, with a reported prevalence of 4.2% in asymptomatic boys and approximately 2% in symptomatic cohorts. TM often coexists with undescended testes, where TM incidence may range from 2.8% to 12%, varicocele, and genetic conditions such as Trisomy 21, with reported prevalence rates ranging from 22.8% to 36%, and McCune-Albright syndrome, with rates up to 62%, although causal links remain uncertain.

Crucially, both articles converge on the fact that isolated TM in children appears largely benign, with extremely few tumors reported in longitudinal pediatric series and meta-analyses. However, pediatric testicular tumors account for 1%–2% of all solid tumors in children, and because a systematic review identified tumors in 3.6% of children with TM, a possible association cannot be completely excluded. These data should be interpreted cautiously, given the heterogeneity of the available studies, potential referral bias, and limited follow-up into adulthood. The main unresolved concerns remain long-term malignancy risk and potential implications for fertility.

## 3 Management, guidelines, and proposed algorithms

Both contributions emphasize a risk-stratified approach. Habachi et al. underline that, in the absence of robust evidence, continued clinical follow-up and education in testicular self-examination are prudent, reserving scheduled ultrasound surveillance for those with additional risk factors. Zitouni et al. compare European (ESUR/EAU) and American (AUA/ACR) guidance: both agree that isolated TM does not require routine imaging; divergence persists for high-risk patients, where European models favor scheduled ultrasound and American guidance prioritizes education and selective imaging.

The Topic therefore supports a pragmatic, risk-adapted approach:

- Isolated TM in children: reassurance, no routine imaging, and patient/caregiver education on monthly self-examination after puberty.
- TM associated with established risk factors, such as cryptorchidism, testicular atrophy, Klinefelter syndrome, or family history of testicular tumors: individualized follow-up including annual physical examinations, urology transition planning, and periodic ultrasound surveillance.

## 4 Unanswered questions and future directions

Authors highlight the paucity of prospective, long-term pediatric cohorts, leaving open whether childhood TM regresses, persists, or truly modifies adult cancer and fertility outcomes. The observation that TM may spontaneously regress in some children and that prevalence varies across symptomatic and asymptomatic cohorts warrants deeper investigation. Both articles call for multicenter, longitudinal studies and for harmonization of guidelines tailored specifically to children rather than extrapolated from adult data.

## 5 Conclusion

Taken together, this Research Topic helps shift the focus of TM in children from a broadly alarming, isolated cancer-linked finding to a mostly benign ultrasonographic marker that requires nuanced, risk-based management rather than blanket surveillance. By clarifying definitions, summarizing epidemiology, and juxtaposing international guidelines, the two reviews provide clinicians with practical direction while clearly defining research priorities: long-term outcomes, fertility impact, and the refinement of follow-up strategies in children identified with TM.

